# Geneva® Series Rootstocks for Apple Trees Under Extreme Replanting Conditions in Southern Brazil

**DOI:** 10.3389/fpls.2021.712162

**Published:** 2021-08-30

**Authors:** Leo Rufato, Pricila Santos da Silva, Aike Anneliese Kretzschmar, Amauri Bogo, Tiago Afonso de Macedo, Juliana Fátima Welter, Gennaro Fazio, Daiana Petry

**Affiliations:** ^1^Department of Agronomy, University of Santa Catarina State, Florianópolis, Brazil; ^2^U.S. Department of Agriculture - Agricultural Research Service (USDA-ARS), Plant Genetic Resources Unit Geneva, Geneva, NY, United States; ^3^Department of Environmental Engineering, University of Santa Catarina State, Florianópolis, Brazil

**Keywords:** apple tree, yield efficiency, replanting area, ‘G.213’, ‘G.210’

## Abstract

Geneva® rootstocks in Brazil are known to be efficient in controlling vigor, and are precocious and resistant to diseases. The objective of this study was to evaluate the performance of apple tree cultivars grafted on the Geneva® rootstocks in severe replant disease areas, by planting 60 days after the eradication. The experiments were implemented in 2017, in São Joaquim and Vacaria. The Gala Select and Fuji Suprema cultivars were grafted onto ‘G.202’, ‘G.814’, ‘G.210’, and ‘G.213’ rootstocks in the Tall Spindle training system. In 2018/2019, total thinning was carried out to promote plant growth. In São Joaquim, partial thinning was carried out in 2019/2020 harvest of ‘Gala Select’. The rootstocks were divided into two groups based on vigor, for both areas and cultivars. ‘G.202’ and ‘G.213’ were 40% less vigorous than ‘G.210’ and ‘G.814’. For ‘Gala Select’, the extreme non-fallow condition mainly affected the vigor and productivity of ‘G.213’ in both areas. At the end of two harvests, ‘G.213’ was 17% less productive than ‘G.210’, contrary to what is observed in areas where the fallow period is respected. However, ‘G.213’ confirmed a greater yield efficiency, which was 27% higher than ‘G.210’. This suggests that a perspective of forecasting production for the third crop is higher for ‘G.213’ than for ‘G.210’. In the case of ‘Fuji Suprema’, the G.210 rootstock was the most productive in both areas. In São Joaquim, ‘G.202’ matched ‘G.210’ in productivity and efficiency as it sprouts better in colder regions. Considering the fruit quality, ‘G.213’ anticipated the maturation with fruits of larger size and higher total soluble solids (TSS) in both areas and cultivars, making it possible to anticipate the harvest. It was concluded that the non-fallow condition does not alter the relative differences in vigor and fruit quality among the rootstocks. However, notwithstanding the overall replant tolerance of these rootstocks, it does reduce productivity by mainly affecting less vigorous rootstocks that need about three crops to overcome the allelopathic effects of the soil and start growing normally. The G.210 semi-dwarfing rootstock is an alternative for the immediate conversion of apple orchards of Gala Select and Fuji Suprema cultivars in southern Brazil.

## Introduction

Most of the modern apple orchards rely on dwarfing rootstocks, which produce a more compact tree allowing for high density planting and earlier and higher yields, and therefore, great economic viability (Afonso et al., 2017). Anticipated production, alternate bearing (Kviklys et al., 2016), resistance and tolerance to pests and diseases (Beers et al., 2006), drought resistance capacity (Tworkoski and Fazio, 2016), resistance to sprouting time, sensory characteristics, and physicochemical composition of fruits (Kviklys et al., 2015) are the other characteristics of apple trees induced by the rootstocks or by the combination of the canopy cultivar and the rootstocks (Harrison et al., 2016).

Currently, in Brazil, there is a gradual change toward the use of densely packed apple orchards with the introduction of the Geneva® series rootstocks (e.g., ‘G.210’ and ‘G.213’) in commercial areas of large companies such as Fischer, Hiragami, Rasip, and Schio. The Geneva® series rootstocks were developed by the apple rootstock breeding program at Cornell University with the original intention of breeding rootstocks resistant to fire blight, collar rot, wooly apple aphid, and replant disorders in addition to size control (Robinson et al., 2011; Fazio et al., 2012). These rootstocks have a wide vigor range, allowing greater plant densities per area and better light inside the canopy (Fazio et al., 2013).

In the south of Brazil, some research has been carried out with the Geneva® series rootstocks ‘G.202’ ‘G.213’, ‘G.210’, and ‘G.814’. According to Denardi et al. (2015), ‘G.213’ has good adaptability and production stability, whereas the G.202 rootstock has less constant productivity over the years. These same authors classified G.202 and G.213 rootstocks as dwarves (vigor similar to M.9). According to Macedo et al. (2019), the Fuji Suprema cultivar grafted on ‘G.213’ is more productive in both virgin and replanting soil. The G.210 rootstock is considered semi-dwarf (Denardi et al., 2015) and has vigor similar to ‘G.814’. According to Pasa et al. (2016), the G.814 rootstock has the potential for use in high-density orchards.

Before choosing the most adapted rootstock, the capabilities and limitations of each rootstock should be evaluated in each growing condition (Gjamovski and Kiprijanovski, 2011). Thus, the objective of this work was to evaluate the productive performance of apple cultivars grafted on the Geneva® series rootstocks under extreme conditions of replanting areas in southern Brazil.

## Materials and Methods

Two experiments with the ‘Gala Select’ and ‘Fuji Suprema’ grafted on Geneva® rootstocks ‘G.202’, ‘G.210’, ‘G.213’, and ‘G.814’ were implemented.

The first experiment was implemented in a commercial orchard of the company named Mareli, located at an altitude of 1,364 m, with the geographic coordinates of 28°16' south latitude and 49°56' west longitude, in São Joaquim, Santa Catarina, Brazil. The soils of the region fall into the classes Cambisolo Humico, Neossolo Litólico, and Nitossolo Háplico, formed from riodacite rock and basalt (Embrapa, 2004). The climate is humid mesothermal type with mild summers, which is Cfb according to the classification of Köppen (Köppen, 1948).

The second experiment was implemented in a commercial orchard of the company Agropecuária Schio Ltda, located at an altitude of 971 m, with the geographical coordinates of 28°24' south latitude and 50°50' west longitude, in Vacaria, Rio Grande do Sul. The climate according to the Köppen classification is Cfb, subtropical with mild summers (Köppen, 1948). The maximum, minimum, and average air temperatures have a marked annual amplitude (Pereira et al., 2009). The soils of the region can be classified as Latossolo Bruno, with smooth to wavy relief, containing high levels of clay and aluminum (Embrapa, 2004).

Both the experiments were implemented in 2017, about 60 days after the eradication of the old orchard, under extreme replanting conditions with no fallow period before planting the trials. The spacing between trees and rows, pruning, training system, and thinning adopted were based on the current grower preference for each region.

In 2018/2019, all fruits were removed during the spring from both the varieties and regions to encourage vegetative growth. In São Joaquim area, in the winter of 2019, pruning was carried out to form and train the trees in the two-dimensional fruit wall system. Partial thinning was carried out in the 2019/2020 harvest, aiming to standardize the production to approximately 7 t.ha^−1^ or 1 (one) fruit per cluster. In Vacaria, in the winter of 2019, minimal pruning was carried out with the sole aim of removing branches that were competitive with the leader centering and training of the trees by the tall spindle system. Maximum plant production was prioritized, with no thinning in 2019/2020. In 2020/2021, no thinning was carried out in both areas.

In São Joaquim area, the trial was planted with a spacing of 0.90 m between trees and 3.2 m between rows (3,472 trees.ha^−1^) for ‘Gala Select’; 1.0 m between trees and 3.2 m between rows (3,125 trees.ha^−1^) for ‘Fuji Suprema’. In Vacaria area, the trial was planted with a spacing of 0.90 m between trees and 4.0 m between rows (2,777 trees.ha^−1^) for ‘Gala Select’; 1.0 m between trees and 4.0 m between rows (2,500 trees.ha^−1^) for ‘Fuji Suprema’.

In 2018/2019, all fruits were removed to encourage vegetative growth. Thus, the analyses accounted for 3 years of vegetative data and two consecutive crops of yield performance and fruit quality. Trunk cross-sectional area (TCSA) (cm^2^), total plant height (m), TCSA increase (cm^2^), canopy volume (m^3^), number of branches, cumulative yield (t.ha^−1^), cumulative yield efficiency (kg.cm^−2^), fruit size (100–120 mm, 120–150 mm, or higher than 150 mm), firmness (*N*), and total soluble solids (TSS) (°Brix) were measured. In both the experiments, we adopted the following methods to measure these variables:

- Trunk cross-sectional area (TCSA) (cm^2^): it was obtained by averaging the longitudinal and transversal measurements of trunk diameter planting line, 10 cm above the grafting point. To transform the diameter values into TCSA, the equation A = (πd^2^)/4 was used, where *d* = trunk diameter.
- Total plant height (m): it was measured with a topographic ruler, from the grafting point to the apex of the plant.
- TCSA increase (cm^2^): the average increase in TCSA was obtained by calculating the average of the differences between subsequent years.
- Canopy volume (m^3^): obtained by measuring L = length, H = height from first branch insertion point to apex, W = width, and using equation (L × H × W) (Macedo et al., 2019).
- Number of branches: it was obtained by counting all the branches larger than 10 cm spreading out from the central leader.
- Cumulative yield (t.ha^−1^): it was obtained by adding the yield of all harvests.
- Cumulative yield efficiency (kg.cm^−2^): it was obtained by adding the yield efficiency of all harvests. The yield efficiency was calculated through the ratio of fruit weight mean per plant (kg.plant^−1^) to crown trunk cross-sectional area (cm^2^), expressed in kg of fruits produced per square centimeter of TCSA.
- Fruit size: a sample of 20 fruits per plot was used to classify the size of the fruits. A ruler with circular holes of different diameters was used to measure the fruits and classify them into three sizes: 100–120 mm (low size), 120–150 mm (intermediate size), and >150 mm (high size). The results were expressed as percentage.
- Firmness (*N*): a sample of 50 fruits per plot was measured with a digital texturometer with 11 mm tip. The measurement was performed in the fruit equatorial zone, after two epidermis discs of about 1 cm in diameter were removed from opposite sides.
- Total Soluble Solids (TSS) (°Brix): it was determined with a digital refractometer from the juice extracted from a slice of each fruit in a sample of 50 fruits.

The experiments were implemented in randomized blocks, using four blocks, four treatments, and five replications. The data were analyzed using univariate and multivariate methods. In the univariate method, they were submitted to F-test and when significant, analyzed by Tukey's test (*p* ≤ 0.05). Before ANOVA, the Shapiro–Wilk and Bartlett tests were performed to analyze normality and homogeneity of variances. Multivariate analysis was performed using principal component analysis (PCA) to analyze the interrelation between variables.

## Results

The results were presented based on two stages. The first stage evaluated the productivity and yield efficiency progression over time. The second stage evaluated the vigor-productivity-efficiency relationship using the final averages of the experiments.

### Area of São Joaquim

**Figure 1:**
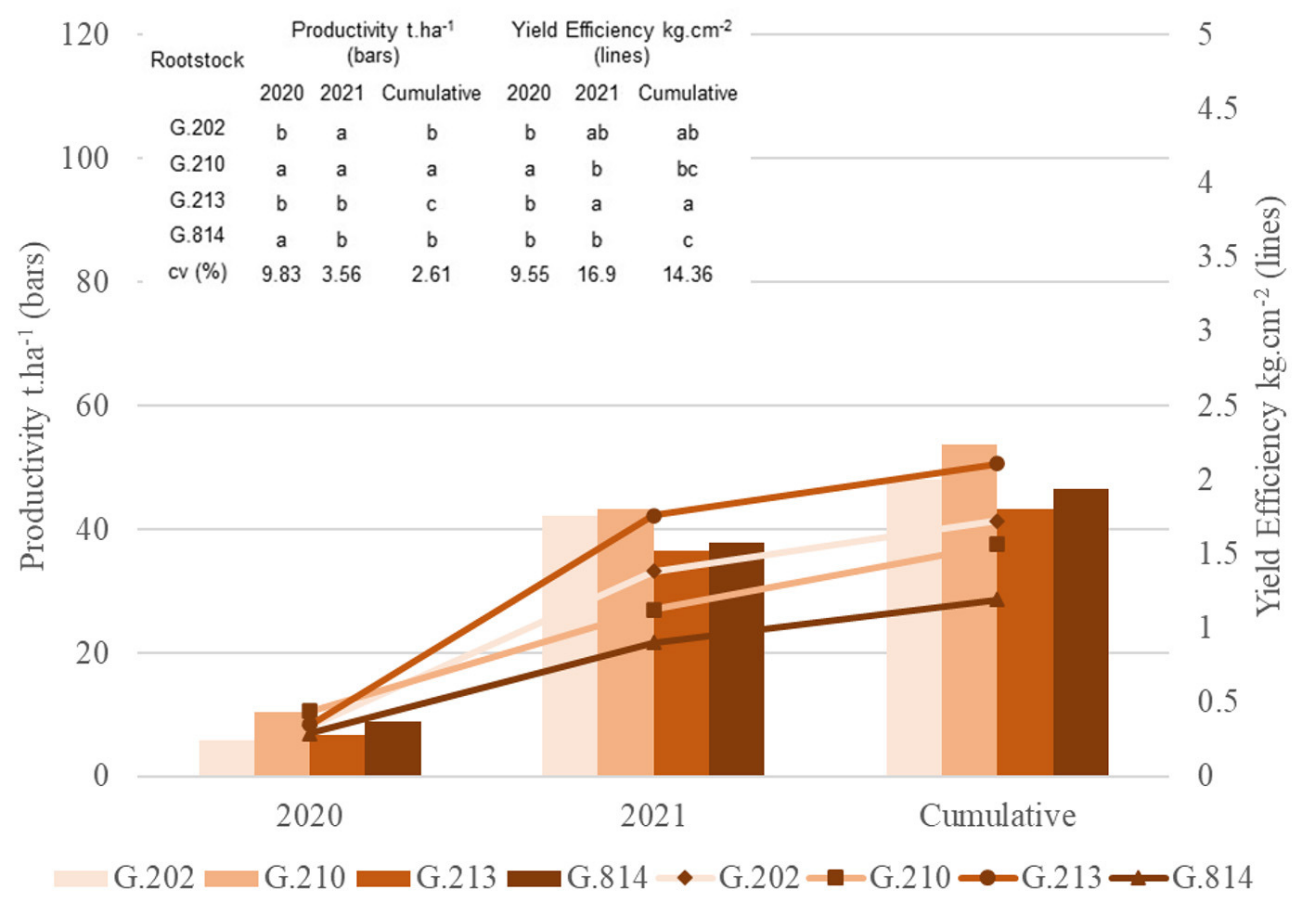
Yield and yield efficiency of the Gala Select cultivar grafted on different rootstocks, under replanting area in São Joaquim. Yield and yield efficiency that do not share a letter are significantly different (Tukey's test, α = 0.05).

In the first harvest (2020), ‘Gala Select’ grafted on ‘G.814’ and ‘G.210’ was more productive than those grafted on other rootstocks. In the second year (2021), the trees on G.202 and G.210 rootstocks had higher productivity. Thus, Gala Select cultivar grafted on the G.210 rootstock had higher accumulated productivity (54 t.ha^−1^) in extreme replanting conditions, 25% higher than the G.213 rootstock (43 t.ha^−1^) and 14% higher than G.814 and G.202 rootstocks (each 47 t.ha^−1^). The trees with greater yield efficiency were grafted on G.213 rootstock, which was 34% higher than the G.210 rootstock (Figure 1).

**Figure 2:**
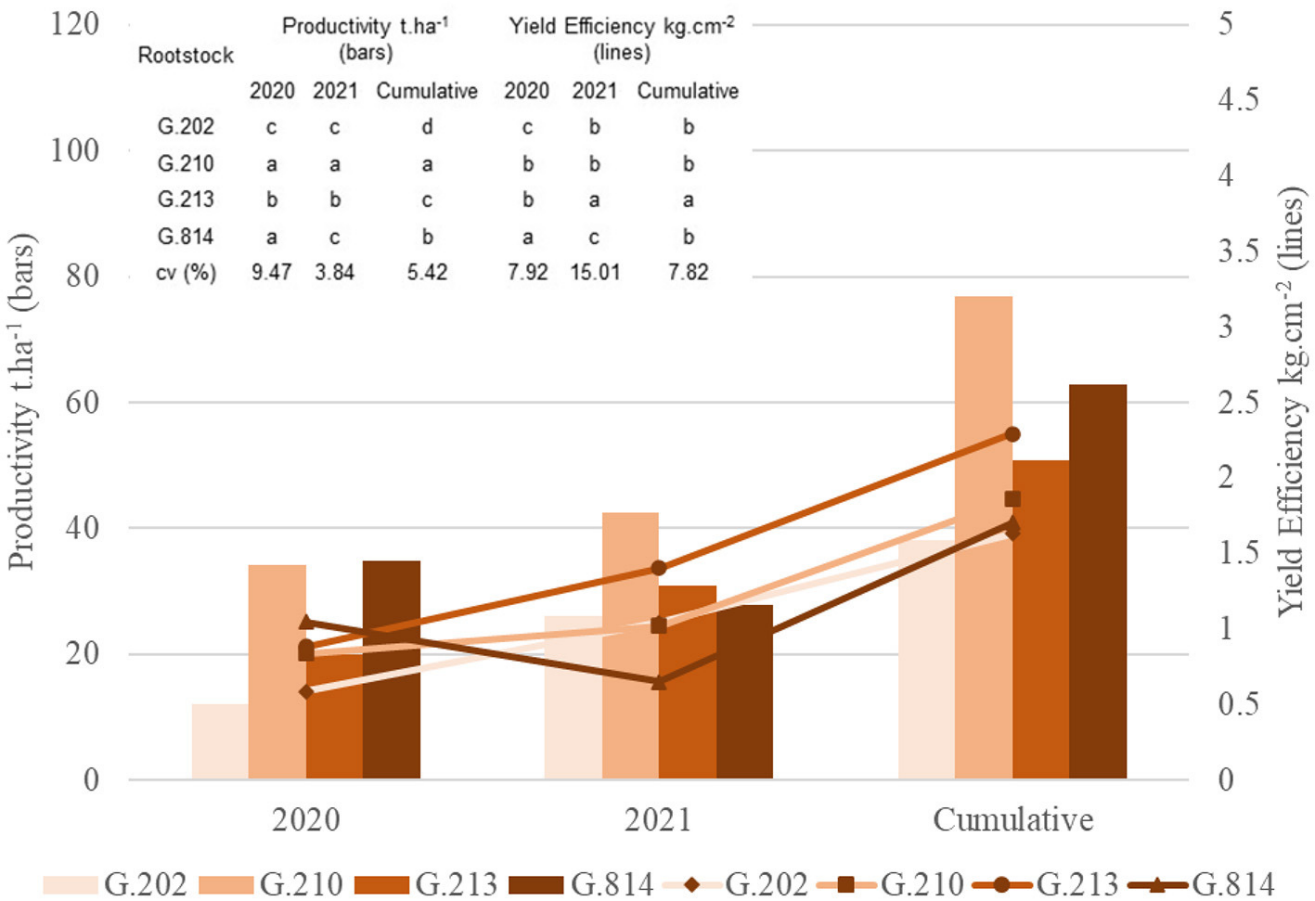
Yield and yield efficiency of the Fuji Suprema cultivar grafted on different rootstocks, under replanting area in São Joaquim. Yield and yield efficiency that do not share a letter are significantly different (Tukey's test, α = 0.05).

For the Fuji Suprema cultivar, high yields were observed on G.814 and G.210 rootstocks in 2020 and on G.210 rootstock in 2021 (Figure 2). Over the period of these 2 years, ‘Fuji Suprema’ on the G.210 rootstock was 102% more productive (77 t.ha^−1^) than on the G.202 rootstock (38 t.ha^−1^). The results of greatest yield efficiency were observed in ‘Fuji Suprema’ on G.213 rootstock.

**Figure 3:**
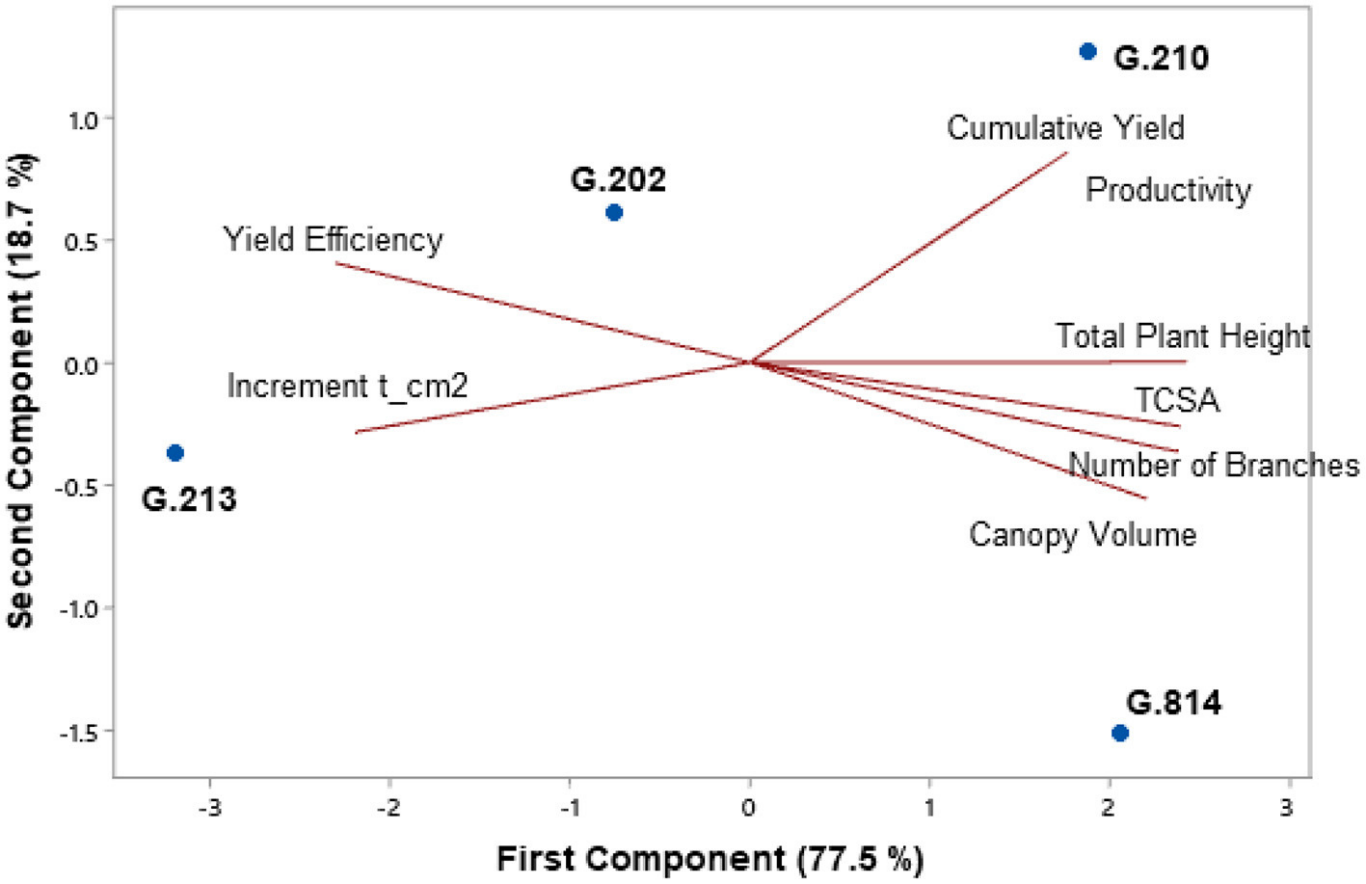
Vigor-productivity-efficiency relationship of the Gala Select cultivar on the Geneva® series rootstocks. São Joaquim – SC.

**Figure 4:**
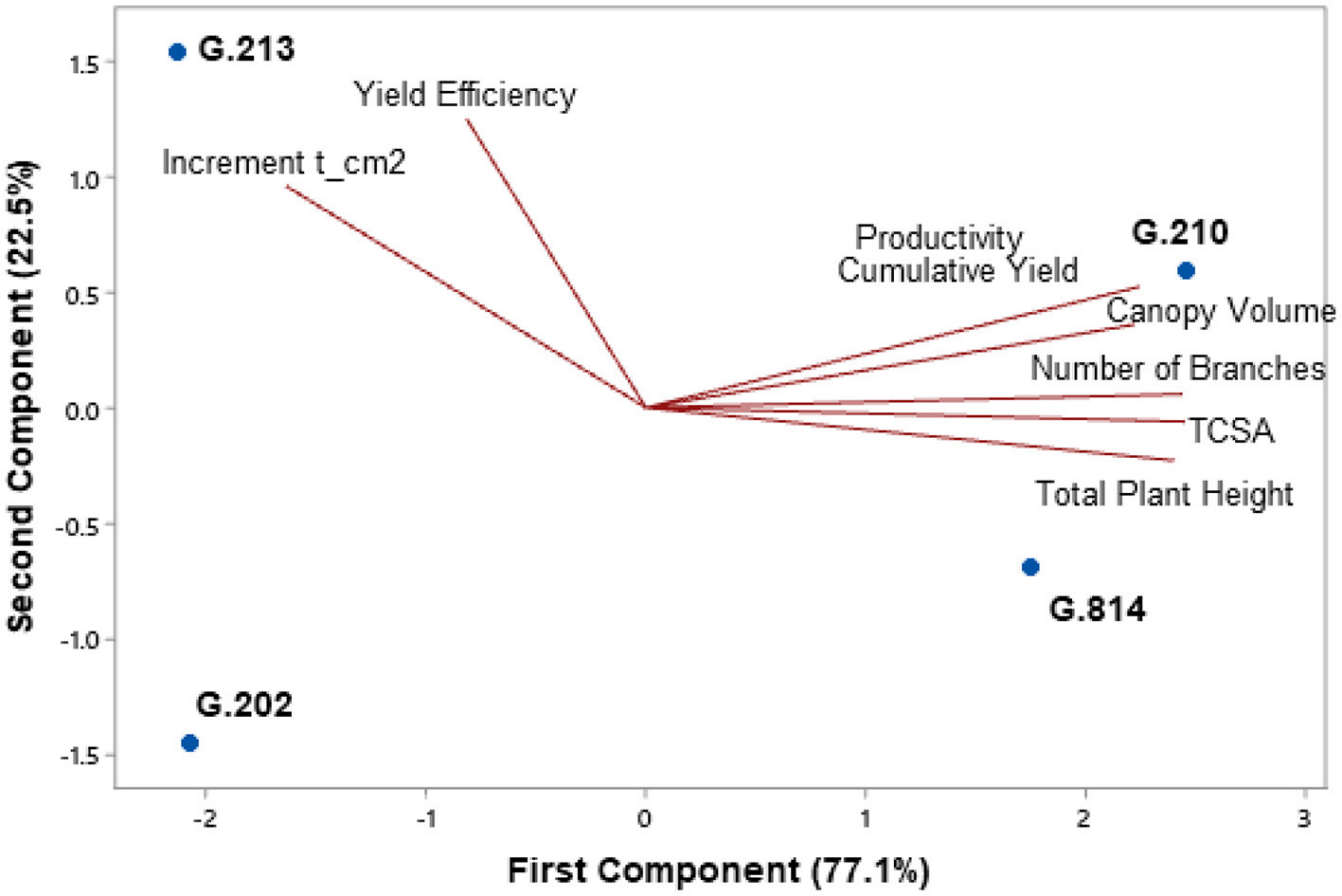
Vigor-productivity-efficiency relationship of the Fuji Suprema cultivar on the Geneva® series rootstocks. São Joaquim – SC.

Figures 3, 4 present the principal component analysis results of the vigor-productivity-efficiency relationship of the Gala Select and Fuji Suprema cultivars. The points represent the rootstocks, and the axes represent the vectors that describe the weight of variables to represent the behavior of the first two main components. Figure 3 shows the results of the Gala Select cultivar and Figure 4 shows that of the Fuji Suprema cultivar.

In both cultivars, the distancing of the dwarfing rootstocks (‘G.202’ and ‘G.213’) from the semi-dwarfing rootstocks (‘G.210’ and ‘G.814’) is observed mainly in terms of vigor. The rootstocks ‘G.202’ and ‘G.213’ were 38.7 and 40% less vigorous than ‘G.210’ and ‘G.814’ in ‘Gala Select’ and ‘Fuji Suprema’, respectively.

In the Gala Select cultivar, the dwarfing rootstocks G.202 and G.213 showed the highest yield efficiency (40%). Between G.202 and G.213 rootstocks there was a difference in relation to productivity. ‘G.202’ correlated with both yield efficiency and productivity, providing greater productivity to the canopy cultivar (11.3%). Despite both being dwarfing rootstocks, ‘G.202’ was about 16.3% more vigorous and 18% less efficient than ‘G.213’. Between the less efficient rootstocks G.210 and G.814, a clear difference in relation to the vigor and productivity of ‘Gala Select’ is shown in Figure 3. ‘G.210’ was more productive (15.3%) and less vigorous than ‘G.814’ (9%).

For the Fuji Suprema cultivar, the yield efficiency did not differentiate the dwarfing rootstocks from the semi-dwarfing ones. In this cultivar, the G.202 rootstock proved to be less efficient and less productive compared to all the others −29% less efficient than ‘G.213’ and 9% less efficient than the semi-dwarfing rootstocks. Thus, ‘G.213’ maintained the characteristic of greater yield efficiency for both cultivars.

The highest yields of the Fuji Suprema cultivar were on the semi-dwarfing rootstocks G.210 and G.814. In this cultivar, ‘G.814’ surpassed the productivity of ‘G.213’ by 23% and was 18% below ‘G.210’. Figure 4 shows a greater correlation between ‘G.210’ and the vigor variables in the Fuji Suprema cultivar compared to that shown in ‘Gala Select’ (Figure 3). The position of ‘G.210’ and ‘G.814’ in relation to the vigor variables indicates the superiority of the G.210 rootstock. In other words, in the Fuji Suprema cultivar in São Joaquim, ‘G.210’ showed greater vigor (8% ASTT) than ‘G.814’.

**Figure 5:**
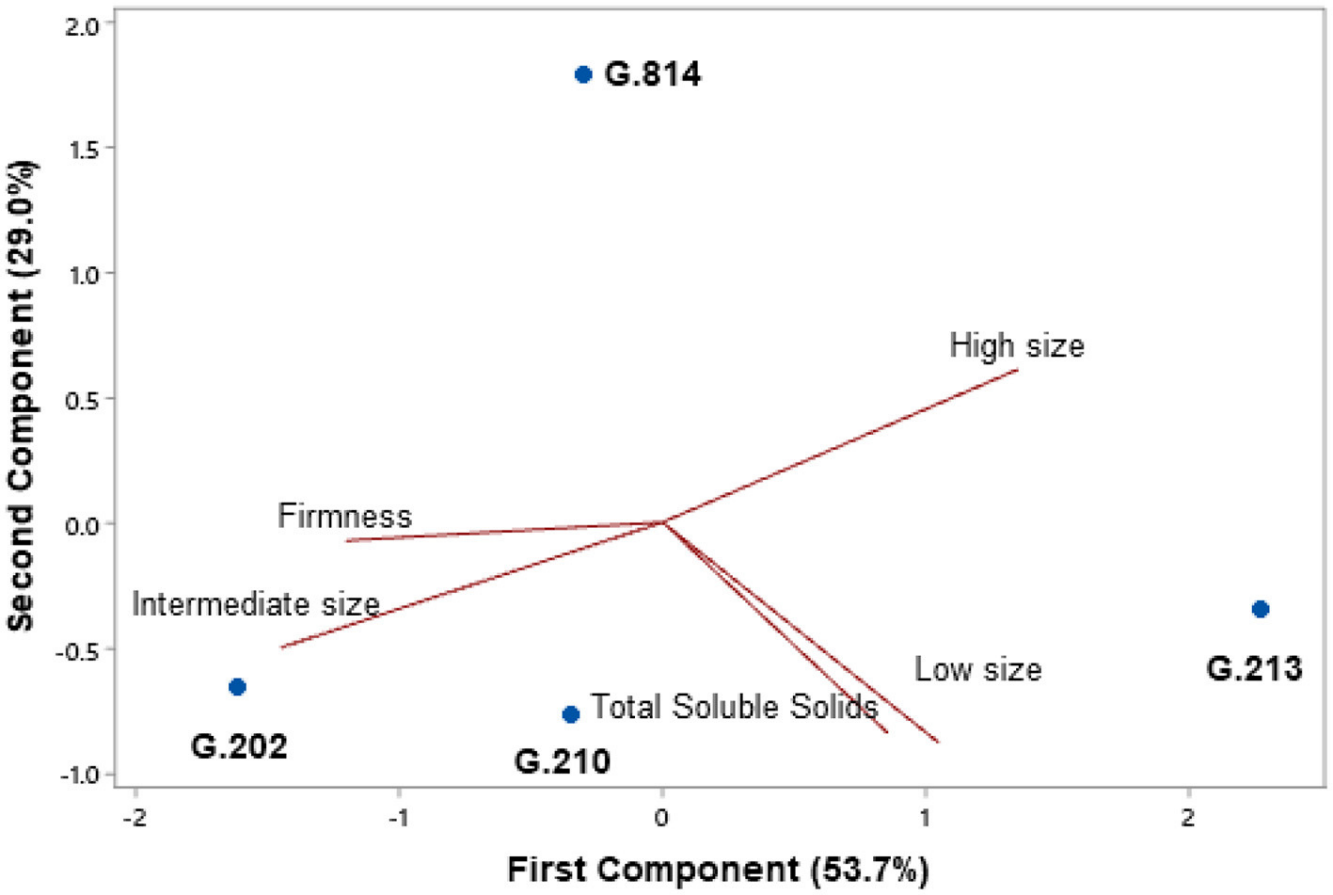
Fruit quality analysis of the Gala Select cultivar on the Geneva® series rootstocks. São Joaquim - SC.

**Figure 6:**
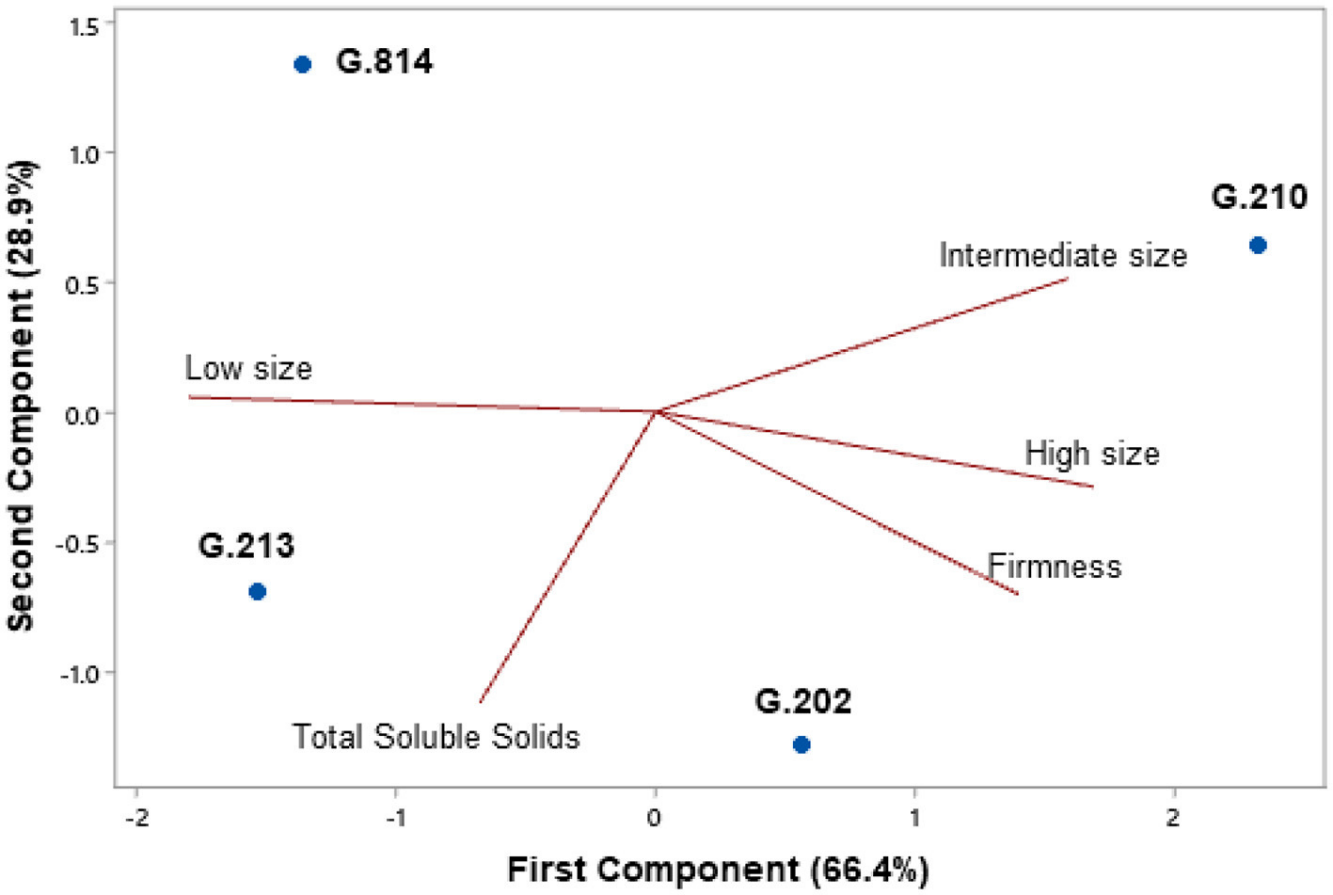
Fruit quality analysis of the Fuji Suprema cultivar on the Geneva® series rootstocks. São Joaquim - SC.

Figures 5, 6 present the principal component analysis results of the fruit quality dimension, for the Gala Select (Figure 5) and Fuji Suprema (Figure 6) cultivars.

Regarding the fruit quality parameters of the Fuji Suprema cultivar, the dwarfing rootstocks differed from the semi-dwarfing rootstocks in having a higher index of total soluble solids. The firmness axis was responsible for differentiating between ‘G.202’ and ‘G.213’. ‘G.202’ showed characteristics of greater fruit firmness than ‘G.213’. All rootstocks provided fruits similarly distributed among the three sizes classes. All of them had the largest number of fruits categorized in the intermediate size class (100% more than in the low size class and 25% more than in the high size class). However, the position of ‘G.210’ on the graph (Figure 6) shows a greater number of fruits in the high size class and lesser in the low size class compared to the other rootstocks.

For the Gala Select cultivar, all rootstocks provided most of the fruits categorized as high size. However, the positions of ‘G.213’ and ‘G.814’ on the graph (Figure 5) identify them with higher amounts of high size fruits compared to ‘G.202’ and ‘G.210’. The difference between ‘G.213’ and ‘G.814’ is on the axis of the low size class. ‘G.213’ has a positive correlation with this axis. In other words, ‘G.213’ has a higher number of fruits categorized as low size when compared to ‘G.814’. ‘G.202’ continued to provide fruits with greater firmness than the other rootstocks. ‘G.210’ resembled ‘G.202’ and ‘G.213’ in total soluble solids.

### Area of Vacaria

**Figure 7:**
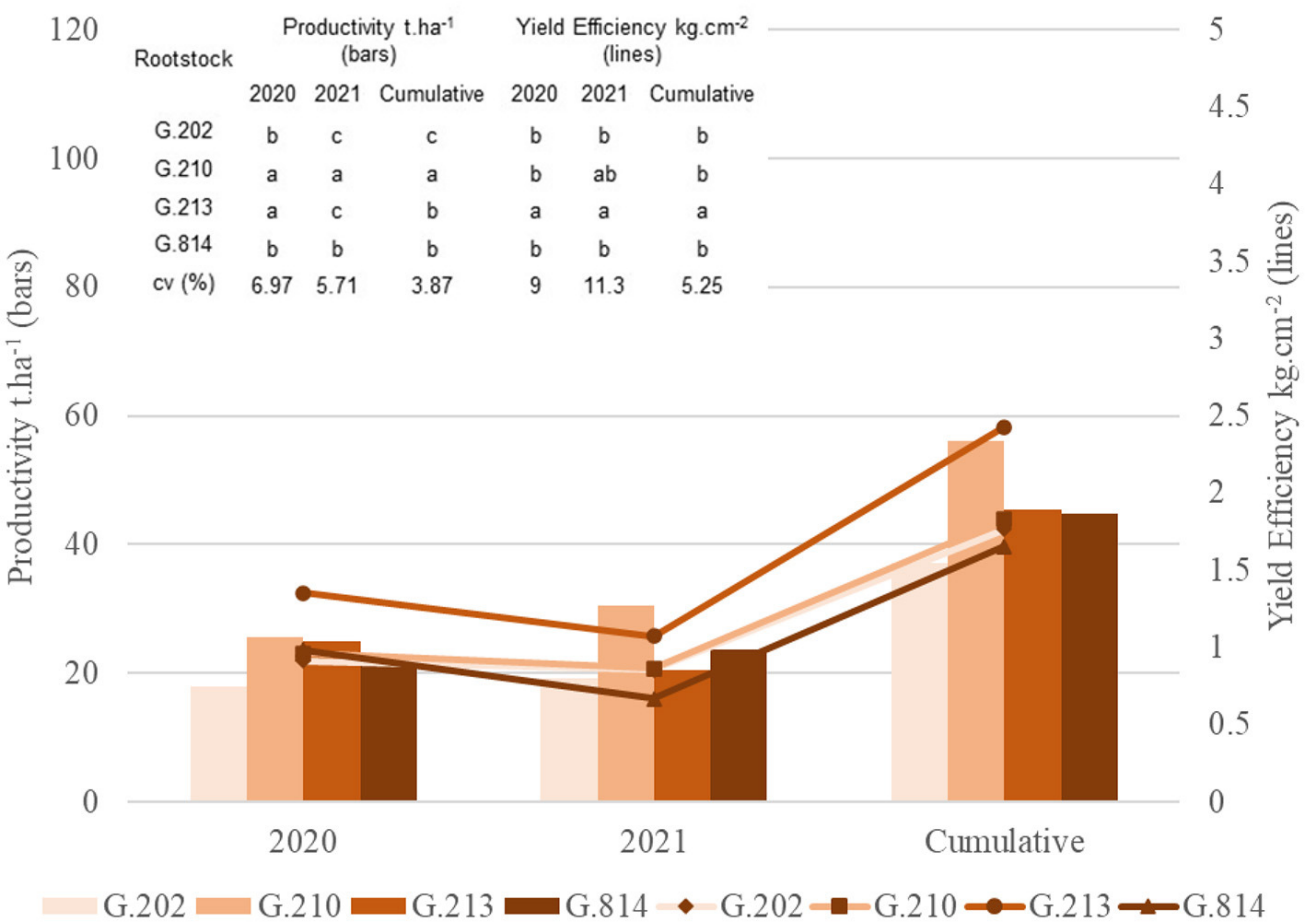
Yield and yield efficiency of the Gala Select cultivar grafted on different rootstocks, under replanting area in Vacaria. Yield and yield efficiency that do not share a letter are significantly different (Tukey's test, α = 0.05).

In the first harvest (2020), the Gala Select cultivar was more productive on the G.210 and G.213 rootstocks (Figure 7). In the second harvest (2021), ‘Gala Select’ grafted on ‘G.210’ had high productivity. In the sum of these 2 years, the Gala Select cultivar on ‘G.210’ was 143% (56 t.ha^−1^) higher than ‘G.202’ (23 t.ha^−1^) and 24% higher than G.814 and G.213 rootstocks (45 t.ha^−1^). However, the G.213 rootstock had a higher yield efficiency than the other rootstocks.

**Figure 8:**
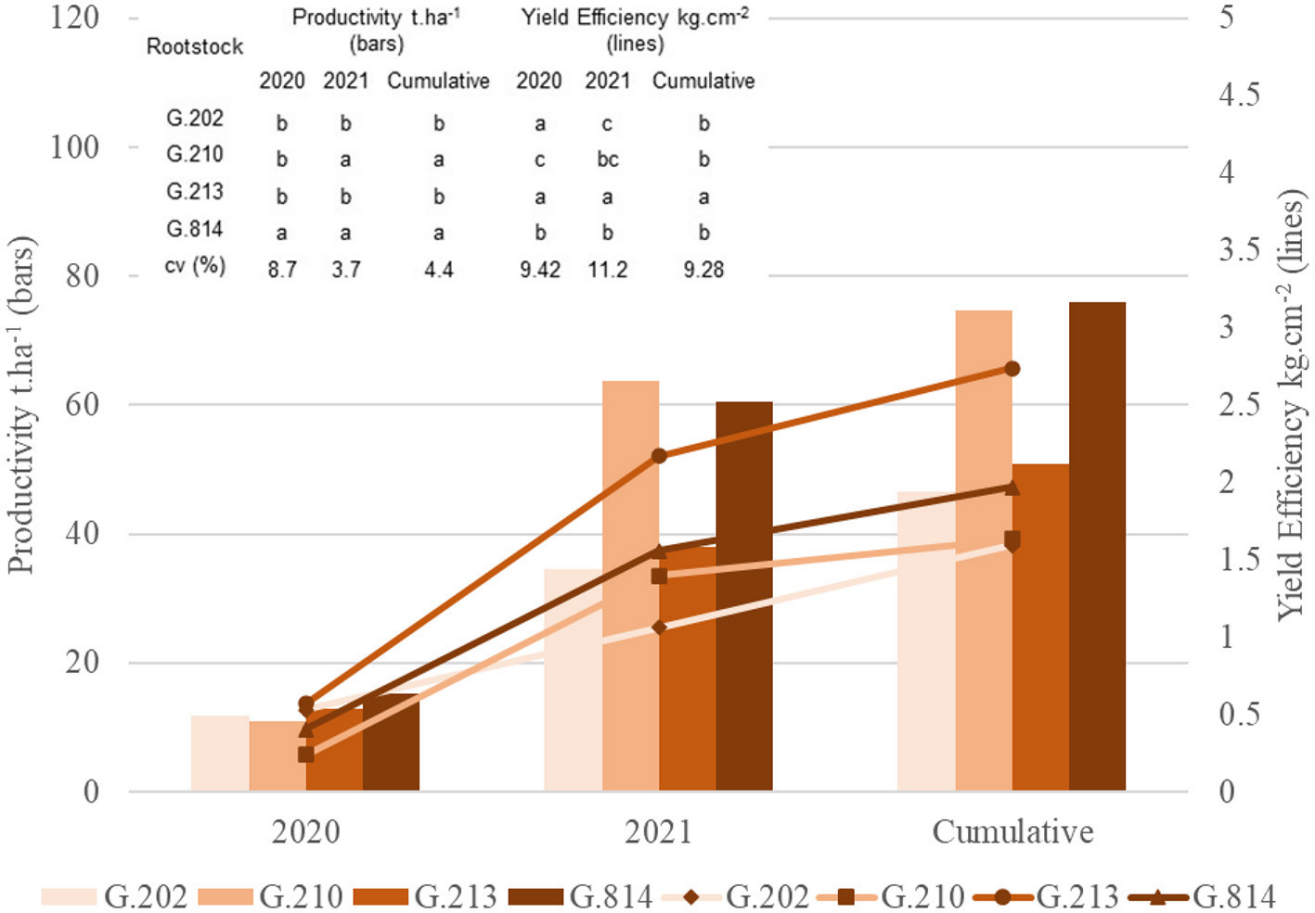
Yield and yield efficiency of the Fuji Suprema cultivar grafted on different rootstocks, under replanting area in Vacaria. Yield and yield efficiency that do not share a letter are significantly different (Tukey's test, α = 0.05).

The Fuji Suprema cultivar had high productivity when grafted on the G.814 rootstock in the 2020 and 2021 harvests, and on the G.210 rootstock in the 2021 harvest (Figure 8). Thus, the cumulative productivity of these 2 years was higher for the G.814 and G.210 rootstocks when compared to the G.202 and G.213 rootstocks. However, even with lower productivity, the trees grafted on ‘G.213’ had a higher yield efficiency than when grafted on other rootstocks.

**Figure 9:**
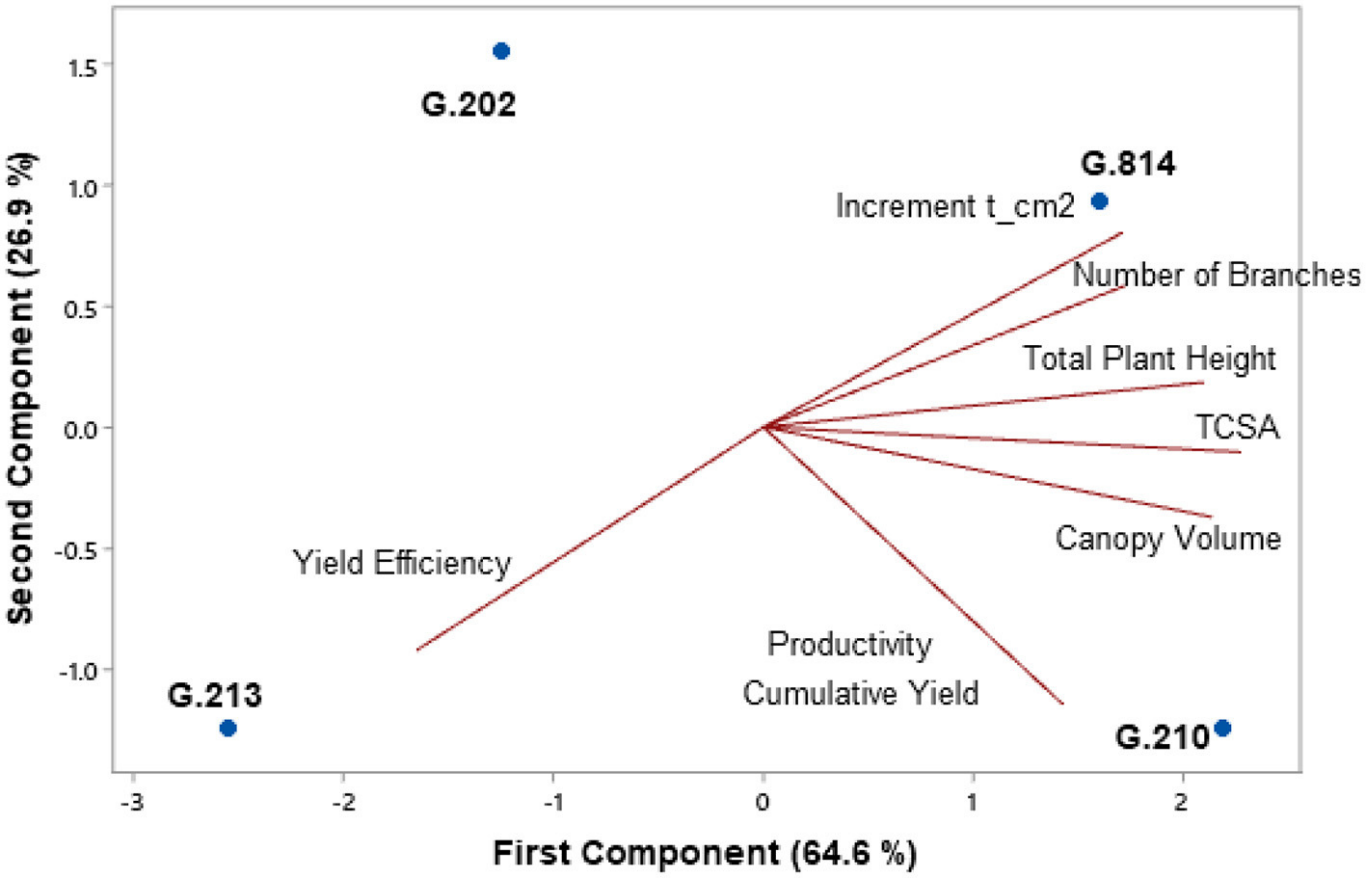
Vigor-Productivity-Efficiency relationship of the Gala Select cultivar on the Geneva® series rootstocks. Vacaria - RS.

**Figure 10:**
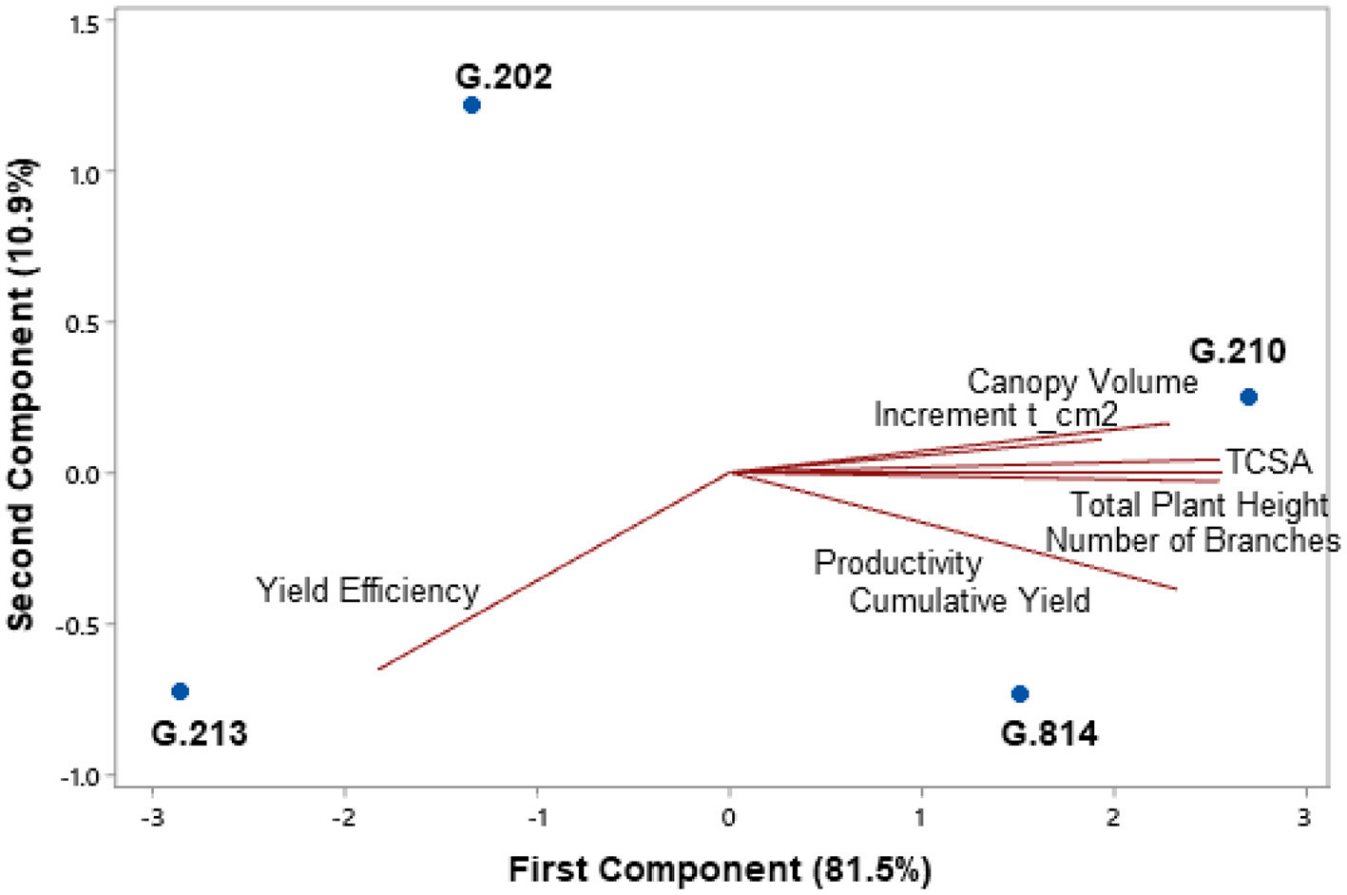
Vigor-productivity-efficiency relationship of the Fuji Suprema cultivar on the Geneva® series rootstocks. Vacaria - RS.

Figures 9, 10 present the principal component analysis results of the vigor-productivity-efficiency relationship of the Gala Select and Fuji Suprema cultivars in Vacaria area. The points represent the rootstocks, and the axes represent the vectors that describe the weight of variables to represent the behavior of the first two main components. Figure 9 presents the results of the Gala Select cultivar and Figure 10 of the Fuji Suprema cultivar.

Based on vigor, there was again the separation of G.814 and G.210 rootstocks from G.202 and G.213 rootstocks for both cultivars. Dwarfing rootstocks were 32 and 43% less vigorous than semi-dwarfing rootstocks for ‘Gala Select’ and ‘Fuji Suprema’, respectively.

‘G.213’ showed higher yield efficiency in both cultivars (43% in ‘Gala Select’ and 32% in ‘Fuji Suprema’). For both cultivars, ‘G.202’ was characterized by low productivity. For the Fuji Suprema cultivar, the semi-dwarfing rootstocks provided higher yields than the dwarfing ones. The greater vigor induced by these rootstocks did not affect the average productivity, and the Fuji Suprema cultivar grafted on ‘G.814’ and ‘G.210’ had 58% higher productivity than when grafted on ‘G.202’ and ‘G.213’ (38 × 24 t.ha^−1^). However, for the Gala Select cultivar, the productivity vector changed direction separating the G.210 and G.814 rootstocks. The G.210 rootstock continued to show higher productivity, and the yield efficiency of ‘G.213’ made it equal to ‘G.814’ in terms of average and accumulated productivity.

**Figure 11:**
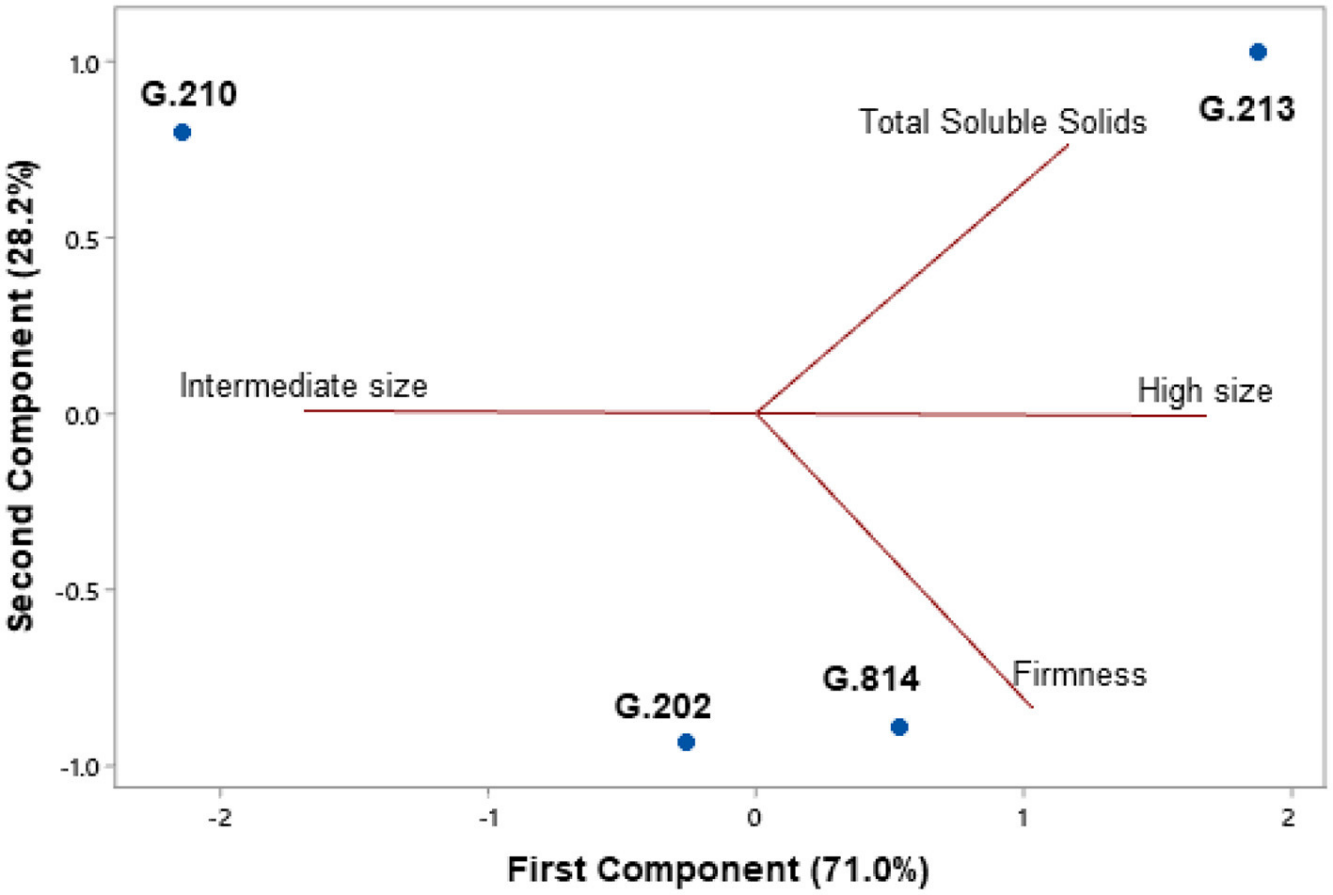
Fruit quality analysis of the Gala Select cultivar on the Geneva® series rootstocks. Vacaria - RS.

**Figure 12:**
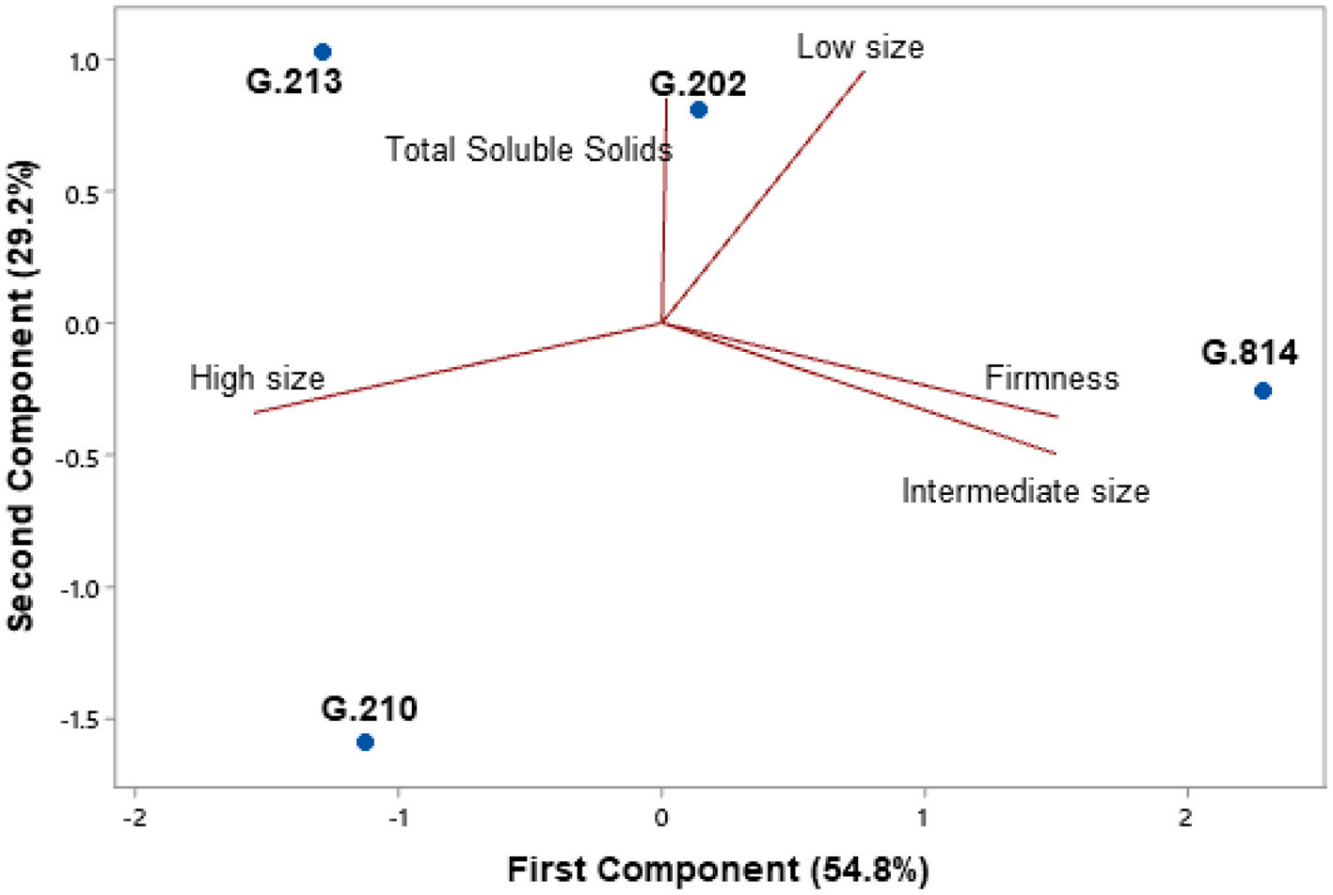
Fruit quality analysis of the Fuji Suprema cultivar on the Geneva® series rootstocks. Vacaria - RS.

Figures 11, 12 present the principal component analysis results of the fruit quality dimension, for the Gala Select (Figure 11) and Fuji Suprema (Figure 12) cultivars.

Two groups can be identified for the Gala Select cultivars. The G.213 and G.210 rootstocks were characterized by higher total soluble solids and lesser firmness when compared to ‘G.202’ and ‘G.814’. The difference in the positions of ‘G.213’ and ‘G.210’ in the graph is due to the fruit size. ‘G.213’ has a greater number of fruits of high size and a smaller number of fruits of intermediary size when compared to ‘G.210’. The G.202 and G.814 rootstocks were characterized by greater firmness and lesser total soluble solids when compared to G.213 and G.210 rootstocks. They were similar in relation to the distribution of fruit size classes, with fruits of high size being greater in quantity than ‘G.210’ and lesser than ‘G.213’. No rootstock showed fruits of the low-size category for the Gala Select cultivar.

For the Fuji Suprema cultivar, the principal component analysis result was similar to São Joaquim area. The total soluble solids axis differentiated the dwarfing rootstocks from the semi-dwarfing rootstocks. ‘G.202’ and ‘G.213’ had higher total soluble solids than ‘G.210’ and ‘G.814’. The G.202 rootstock showed characteristics of greater firmness than the G.213 rootstock. The size and firmness axes were responsible for separating ‘G.210’ from ‘G.814’. The G.210 rootstock provided a larger number of fruits with greater size than the G.814 rootstock. ‘G.814’ resulted in more firm fruits than ‘G.210’. The G.213 rootstock showed a strong positive correlation with the classes of high- and low-size fruits. Thus, the quantity of high-size fruits provided by ‘G.213’ was similar to that provided by ‘G.210’; however, ‘G.213’ provided a greater quantity of low-size fruits when compared to ‘G.210’.

## Discussion

In both the cultivars and experimental areas, it was possible to divide the rootstocks into two groups based on vigor. The G.814 and G.210 rootstocks were allocated to the group of rootstocks that induced greater vigor (semi-dwarfs) to the canopy cultivars and the G.202 and G.213 rootstocks formed the group of rootstocks that induced smaller vigor (dwarfs) to the cultivars. The dwarfing rootstocks were ~38% less vigorous than the semi-dwarfing rootstocks. The classification of ‘dwarf rootstock’ is related to the efficient control of the vigor of trees induced by rootstock (Denardi et al., 2015). Thus, it is possible to claim that even under extreme replanting conditions the evaluated Geneva rootstocks retained the same vigor characteristics as noted by Denardi et al. (2016) and Macedo et al. (2021) in replanting areas.

In São Joaquim and Vacaria, the ‘Gala Select’ apple trees in the G.210 rootstock had high accumulated productivity (2 years), and hence these trees were able to express a high productive potential. Both ‘G.210’ and ‘G.814’ were categorized as semi-dwarfs; however, the lower vigor of ‘G.210’ compared to ‘G.814’ made it more efficient than ‘G.814’. Thus, in extreme replanting conditions, greater vigor combined with yield efficiency seems to be related to the productive performance of apple trees. The high accumulated productivity of the G.210 rootstock was also observed by Reig et al. (2020) in an experiment developed in a replanting area in the United States of America (USA), using Super Chief Delicious cultivar.

For the Gala Select cultivar in São Joaquim, the non-fallow condition reduced the productivity of the trees on all rootstocks, especially on ‘G.213’, which showed 17% less productivity at the end of two harvests when compared to ‘G.210’ (the most productive). In Vacaria area, due to the lack of thinning in 2019, yields started at a higher level than in the São Joaquim area, resulting in a low increase in production for all rootstocks in the next harvest. The G.213 rootstock was the most affected as it showed less vigor. According to Denardi et al. (2018), on replanting soil conditions, the performance of apple trees may vary according to the rootstock that is used. In general, the most vigorous rootstocks are less affected by apple replant disease when compared to the dwarfing ones, and the tolerance of their root system to harmful agents has a crucial role to play (Isutsa and Merwin, 2000; Leinfelder and Merwin, 2006).

Macedo et al. (2021) evaluated the productive performance of the Gala Select cultivar grafted on Geneva ‘G.213’ and on rootstocks currently used in southern Brazil (M.9 and Maruba/M.9) in virgin and replanting areas. In the replanting area, fallow period of 12 months was grown before conversion of the orchard. The mean productivity of ‘G.213’ over a period of 4 years was around 21 t.ha^−1^ in both areas, which was very similar to the value of 22 t.ha^−1^ found in our study (Table 1). The cumulative yield in virgin area was higher than in the replanting area over the period of 4 years. However, virgin areas are no longer widely available. Thus, it is important to assess the real need for fallow periods before the orchards are converted. The average and accumulated yields of the replanting area with fallow were numerically smaller than our results obtained for the replanting area without fallow (extreme replanting conditions). Both replanting areas took a year longer than the virgin area to start production. This result shows that trees grafted mainly on dwarfing rootstocks require more time to reach vegetative conditions sufficient for the expected productivity in replanting areas.

**Table 1:** Yield of the Gala Select cultivar grafted on ‘G.213’, in the virgin area, the replanting area with fallow, and the replanting area without fallow in Vacaria.

	**Implantation – 2011**		**Implantation - 2017**	
**Year**	**Virgin soil**	**Replanting area** **(with fallow)**	**Year**	**Extreme replanting area** **(without fallow)**
2012	–	–	2018	–
2013	3.2	–	2019	-
2014	30.6	26.6	2020	24.88
2015	29.4	16.5	2021	20.46
**Mean**	**21.07**	**21.55**	**Mean**	**22.67**
**Sum**	**63.2**	**43.1**	**Sum**	**45.34**
2016	44.6	45.9		
2017	57.5	38.4		
2018	67.7	58.6		
2019	69.2	37.9		
2020	45.2	44.8		
**Mean**	**43.43**	**38.39**		
**Sum**	**347.4**	**268.7**		

*Results related to virgin area and replanting area with fallow were published by Macedo et al. (2021). Results related to the replanting area without fallow (extreme replanting conditions) were obtained from the present work*.

However, the 12-month fallow replanting area remained without apple cultivation for 12 months, which is 1 year longer than for the replanted area without fallow. Thus, the productivities obtained from this work in comparison with those published by Macedo et al. (2021) suggests the possibility of immediate conversion of apple orchards of the Gala Select cultivar grafted onto the Geneva G.213 rootstock.

The mean yield efficiency of ‘G.213’ was around 1.12 in the trials by Macedo et al. (2021), while that obtained from this work was 1.14. In other words, ‘G.213’ maintained its yield efficiency even under extreme replanting conditions. Thus, it is noteworthy that the main characteristics of these rootstocks, such as yield efficiency and production stability, remain unchanged even under extreme replanting conditions. Macedo et al. (2021) also highlighted that the G.213 rootstock had the lowest alternation bearing in replanting soil, making it a rootstock of high and stable productivity.

The Gala Select cultivar on the G.213 rootstock formed trees with greater yield efficiency than when grafted on ‘G.210’, in São Joaquim and Vacaria. According to Robinson et al. (2006), some pieces of evidence affirm the direct relationship between yield efficiency and the ananizing effect of rootstocks. The average increment of production per increment of TCSA of ‘G.213’ was 50.83 tons, while that of ‘G.210’ was 6.03 tons. In other words, for a small difference in TCSA from 1 year to the next, ‘G.213’ increased its production by many more tons when compared to ‘G.210’. In Vacaria, due to the superiority of yield efficiency (30% higher than ‘G.210’), the difference in productivity between ‘G.213’ and ‘G.210’ (more productive) was ~18%. This result corroborates with Rufato et al. (2015) and Macedo et al. (2021), who observed greater yield efficiency among apple trees grafted on G.213 rootstock in replanting areas, based on an experiment developed in Vacaria.

In São Joaquim area, ‘Fuji Suprema’ grafted on the G.210 rootstock produced trees with high accumulated productivity within 2 years of harvest, followed by the G.814 rootstock. In Vacaria, the Fuji Suprema cultivar in G.814 and G.210 rootstocks produced trees that were equally more productive in the same 2 years. The G.213 rootstock produced trees with lower productivity and greater yield efficiency. However, the yield efficiency of the G.213 rootstock compared to semi-dwarf rootstocks at ‘Fuji Suprema’ was 39% less than at ‘Gala Select’. This relationship explains the lower productivity of the Fuji Suprema cultivar by the G.213 rootstock, because the Fuji Suprema cultivar is more vigorous and is better adapted to more vigorous rootstocks than the Gala Select cultivar (Denardi et al., 2018).

From the results obtained on the accumulated productivity of 2 years, it was possible to infer that the dwarf rootstocks G.202 and G.213 were not able to express all their productive potential for the Gala Select cultivar, under extreme conditions of replanting. The replanting conditions delayed the development of dwarf rootstocks, especially the G.213 rootstock, which required more time to provide the expected productive performance for the Gala Select cultivar. One of the characteristics of dwarf rootstocks is the precocious production. According to Fazio and Robinson (2019), the dwarfing Geneva® rootstocks are much more precocious, with flowering in the first year in an orchard or even in a nursery. However, under extreme conditions of replanting, the expected precocity was not observed. In this condition, ‘G.210’ produced more than ‘G.213’ accumulated in the first two crops. However, ‘G.213’ maintained its characteristic of yield efficiency, with 39% less vigor and 37% more yield efficiency than G.210. As described by Robinson et al. (2011), it is also necessary to consider that greater vigor can result in a laborious orchard.

For the Fuji Suprema cultivar, the effect of the non-fallow condition was less noticeable when comparing the productive performance on the rootstocks analyzed. This is because the Fuji Suprema cultivar already showed higher productivity when grafted on semi-dwarf rootstocks (Pasa et al., 2017). Thus, the more vigorous rootstocks G.210 and G.814 remain important alternatives for the productivity of the Fuji Suprema cultivar, even under extreme replanting conditions.

As for the fruit quality, the G.213 rootstock provided sweeter fruits in both areas and cultivars, indicating the characteristic of possible harvest anticipation. As for the fruit size, the G.213 dwarf rootstock provided a greater quantity of high-size fruits in the Gala Select cultivar and the semi-dwarf G.210 rootstock in the Fuji Suprema cultivar. The fruit size of the Gala Select cultivar provided by G.213 is in accordance with Denardi et al. (2015), who compared G.213 and G.210 rootstocks and concluded that the greatest ‘Gala Select’ fruit weight was by the G.213 rootstock. However, Pasa et al. (2017) evaluated the fruit size in the Fuji Suprema cultivar for different rootstocks and concluded that the differences in fruit weight are not consistent along the years and are probably not due to rootstock influence.

In general, the fallow time normally respected in the process of reconverting apple orchards has always been considered essential to guarantee the complete development of rootstocks and canopy cultivars. However, this fallow time economically results in the loss of a production crop. In both experimental areas, it was necessary to postpone the first harvest and carry out total thinning to allow the vegetative development of the trees. Thus, despite the immediate conversion, the orchards that did not have a fallow period took the same time to start production as if they had gone through the fallow period. Compared to the virgin area, notwithstanding the overall replant tolerance of Geneva series rootstocks, the non-fallow reduced the productivity by mainly affecting the less vigorous rootstocks that needed about three crops to overcome the allelopathic effect of the soil and start growing normally. However, on comparing the replanting areas (with and without fallow), it was found that both took the same time to start production after implementation and had the same accumulated productivity in 4 years. However, the one with fallow remained a year without any cultivation. Thus, it can be stated that immediate conversion, without fallowing, can be considered as a strategy in the absence of virgin area for the implantation of new orchards.

Considering the goal of 150 tons in five harvests to cover the costs of orchard implanting, in non-fallow conditions, the G.210 semi-dwarf rootstock was considered to be a good alternative for Gala Select cultivar. Taking into account that the productive potential tends to increase in the next harvest and considering an average of 27.4 tons in the first two harvests, the prospect of reaching the goal was feasible. For the Fuji Suprema cultivar, the G.210 rootstock was also the one that best adapted to the extreme replanting conditions. Productivity was also reduced; however, it resulted in an average of 37.8 tons, ensuring that the goal in five harvests is easily exceeded.

Thus, the immediate conversion leads to the need for 2 years for vegetative formation of the trees and reduces productivity in the first harvest. However, even with reduced productivity in the first harvest, the G.210 rootstock proved to be capable of achieving a productivity of at least 150 tons in five harvests.

## Conclusions

The immediate reconversion of the orchards without fallowing, results in 2 years without vegetative formation for the trees. With or without fallow, the time that elapsed from the orchard implantation until the first commercial harvest remained the same. In replanting area with fallow, the time that elapsed from the orchard eradication until the first commercial harvest was 1 year longer than replanting areas without fallow (extreme replanting conditions).

The non-fallow condition does not alter the difference in vigor and fruit quality between the rootstocks. However, it results in lower productivity, mainly affecting less vigorous rootstocks that need about 3 years to overcome the allelopathic effect of the soil and start growing normally.

The G.210 rootstock is an alternative for the immediate conversion of apple orchards of Gala Select and Fuji Suprema cultivars, in southern Brazil.

## Data Availability Statement

The raw data supporting the conclusions of this article will be made available by the authors, without undue reservation.

## Author Contributions

LR carried out conceptualization, methodology, validation, supervision, project administration, and funding acquisition. PS was involved in investigation and validation. AK was involved in writing–original draft preparation and resources. AB carried out investigation and data curation. TM contributed to the methodology. JW carried out the investigation. GF contributed to the methodology, as well as the writing, reviewing, and editing of the draft. DP was involved in the writing, reviewing, and editing of the draft, as well as the visualization and formal analysis. All authors contributed to the article and approved the submitted version.

## Conflict of Interest

The authors declare that the research was conducted in the absence of any commercial or financial relationships that could be construed as a potential conflict of interest.

## Publisher's Note

All claims expressed in this article are solely those of the authors and do not necessarily represent those of their affiliated organizations, or those of the publisher, the editors and the reviewers. Any product that may be evaluated in this article, or claim that may be made by its manufacturer, is not guaranteed or endorsed by the publisher.
